# Gaze-cued shifts of attention and microsaccades are sustained for whole bodies but are transient for body parts

**DOI:** 10.3758/s13423-022-02087-z

**Published:** 2022-04-05

**Authors:** Nicole X. Han, Miguel P. Eckstein

**Affiliations:** 1 Department of Psychological and Brain Sciences, University of California, Santa Barbara, USA; 2 Department of Psychological and Brain Sciences, Institute for Collaborative Biotechnologies, University of California, Santa Barbara, USA

## Abstract

Gaze direction is an evolutionarily important mechanism in daily social interactions. It reflects a person’s internal cognitive state, spatial locus of interest, and predicts future actions. Studies have used static head images presented foveally and simple synthetic tasks to find that gaze orients attention and facilitates target detection at the cued location in a sustained manner. Little is known about how people’s natural gaze behavior, including eyes, head, and body movements, jointly orient covert attention, microsaccades, and facilitate performance in more ecological dynamic scenes. Participants completed a target person detection task with videos of real scenes. The videos showed people looking toward (valid cue) or away from a target (invalid cue) location. We digitally manipulated the individuals in the videos directing gaze to create three conditions: whole-intact (head and body movements), floating heads (only head movements), and headless bodies (only body movements). We assessed their impact on participants’ behavioral performance and microsaccades during the task. We show that, in isolation, an individual’s head or body orienting toward the target-person direction led to facilitation in detection that is transient in time (200 ms). In contrast, only the whole-intact condition led to sustained facilitation (500 ms). Furthermore, observers executed microsaccades more frequently towards the cued direction for valid trials, but this bias was sustained in time only with the joint presence of head and body parts. Together, the results differ from previous findings with foveally presented static heads. In more real-world scenarios and tasks, sustained attention requires the presence of the whole-intact body of the individuals dynamically directing their gaze.

## Introduction

Orienting attention to prioritize the most relevant scene regions is essential to find an object or person in a cluttered visual environment. Studies with simple arrows or boxes predicting a target’s location have led to advances in the understanding of attention’s performance benefits (Carrasco, 2006, 2011; M. P. Eckstein et al., 2013; Luck et al., 1994; Posner, 1980), mediating computational models (Carrasco, 2006; Dosher & Lu, 2000; M. P. Eckstein et al., 2002, 2004), and neural mechanisms (Carrasco, 2006; Corbetta & Shulman, 2002; Gandhi et al., 1999; Garcia et al., 2013; Giesbrecht et al., 2003; Pestilli & Carrasco, 2005).

In everyday visual search, humans optimize their performance by orienting their attention, not towards arrows and boxes, but towards scene properties and objects that might indicate the probable location of a sought object (Castelhano & Heaven, 2011; M. Eckstein, 2017; M. P. Eckstein et al., 2006; Koehler & Eckstein, 2017a; Malcolm & Henderson, 2010; Võ et al., 2019). A person’s gaze is an important cue in real-world scenes indicating possible future actions and points of interest (Emery, 2000; Kleinke, 1986). Humans, as early as 10 months old (Brooks & Meltzoff, 2005), and other animals (Bräuer et al., 2005; Bugnyar et al., 2004) use gaze direction to orient attention. Humans are good at perceiving others’ gaze directions in daily social interactions (Hessels, 2020). Experiments with gaze cues show signatures of attentional shifts and performance facilitation even when the cue is non-predictive. This involuntary orientation of attention is known as exogenous attention (Egeth & Yantis, 1997; Jonides & Jonides, 1981; Mulckhuyse & Theeuwes, 2010) in contrast to voluntary endogenous attention triggered by central arrows (Cheal & Lyon, 1991; Müller & Rabbitt, 1989; Posner & Cohen, 1984). However, gaze cues also show signatures of sustained attention, with the facilitation appearing as early as 100 ms and lasting up to 300–500 ms after the gaze cue onset and then decaying afterward. The sustained effect of gaze cues lasts longer than a peripherally-presented exogenous synthetic cue for which facilitation disappears at 300 ms (Driver et al., 1999; Friesen et al., 2004; McKee et al., 2007; Posner & Cohen, 1984; Ristic et al., 2007; Theeuwes, 1991).

One limitation of most previous experiments is that they have not captured the dynamics of the various body cues (gaze, head, body) during natural gazing behavior. The studies have used either static images of faces with various gaze and or body directions (Azarian et al., 2017; Bayliss et al., 2004; Driver et al., 1999; Friesen & Kingstone, 1998), highly simplified dynamic figures such as moving point-lights (Shi et al., 2010; Wang et al., 2014), videos of a single individual’s face or animations (Hermens & Walker, 2012; Kuhn & Tipples, 2011). One recent study has applied a realistic environment with dynamic human avatars’ gaze cues (Gregory, 2021). In addition, the gaze cue effects have been typically evaluated with detection tasks of simple stimuli such as letters, dots, and asterisks (Azarian et al., 2017; Driver et al., 1999; Hietanen, 1999; Kingstone et al., 2000), or more complex localization or discrimination tasks (Gregory & Jackson, 2021), rather than more ecologically valid tasks in real scenes.

More realistic scenarios involve individuals directing gaze that appear away from an observer’s fovea and various distances from the observer, making it difficult to infer gaze direction from the visual periphery (Loomis et al., 2008; Palanica & Itier, 2017). These realistic scenarios involve the integration of multiple dynamic cues: gaze (Azarian et al., 2017; Bayliss et al., 2004; Frischen et al., 2007; Hietanen, 1999, 2002), head orientation (Hessels, 2020; Langton et al., 2004; Mareschal et al., 2013; Otsuka, 2014) and body postures (Azarian et al., 2017; Shi et al., 2010; Wang et al., 2014; Zhao et al., 2014).

Our goal was to investigate how people’s natural gaze behaviors contribute to the orienting of covert attention in a complex, ecologically relevant search task with videos of natural dynamic scenes. To our knowledge, this is the first study that embeds the task in dynamic videos of the real world and explores gaze orienting individuals at various retinal eccentricities and viewing distances.

We focused on viewing angles of faces subtended by people located in the real 3D world at mid to long-range distances (5–30 m) from the observer. We aim to understand how the individual head, body motion, and joint presence contribute to orienting attention, influence search performance, and understand the temporal dynamics of attention for each bodily cue. We used video clips recorded while we instructed multiple actors to look to a specified location in the scene that would subsequently present a target person (50% cue validity) among distractor individuals in different complex dynamic scenes. We created different conditions by erasing heads or bodies of only the gaze orienting individuals using an algorithm that eliminated head or body features and replaced them with the background. To understand whether the orienting of attention with the head/body cues is transient or sustained, we utilized two different delay periods (200 ms and 500 ms) before the target's appearance. We measured the effects of the head, body, or joint direction validity (cueing effect) on the accuracy of detecting the target person. In addition, we measured how the head/body direction influenced the direction of microsaccades. Previous studies have found that microsaccade rates, latency, and directions are highly correlated with the spatial cueing direction of covert attention (Engbert & Kliegl, 2003; Hafed & Clark, 2002; Meyberg, Sinn, et al., 2017; Meyberg, Sommer, et al., 2017; Pastukhov & Braun, 2010; Poletti et al., 2017). The majority of the microsaccade studies have used static cues such as central arrows (Engbert & Kliegl, 2003; Meyberg et al., 2017; Meyberg, Sommer, et al., 2017) or simple shapes or flashes present in the periphery (Hafed & Clark, 2002; Laubrock et al., 2005). Here, we assessed how microsaccades are influenced by the dynamic head, body, and joint orienting and compared their modulation to the influences on behavioral performance.

## Material and methods

### Subjects

Thirty undergraduate students (aged from 18–21, 18 females, 12 males) from the University of California Santa Barabra were recruited as subjects for credits in this experiment. All have normal to corrected-to-normal vision. All participants signed consent forms to participate in the study. The sample size was selected based on previous studies on gaze cues, which usually range from 20 to 30 (Friesen & Kingstone, 1998; Palanica & Itier, 2015; Yokoyama & Takeda, 2019).

### Experimental setup and stimuli

All stimuli were presented at the center of the computer screen, subtending a visual angle of 18.4˚ × 13.8˚ (width × height). Participants’ eyes were 75 cm from the computer screen with the head positioned on a chin rest while watching the videos. Each subject’s left eye was tracked by a video-based eye tracker (SR Research EyeLink 1000 plus Desktop Mount) with a sampling rate of 1000 Hz. Subjects completed a calibration and validation routine before each experimental session. If a large eye drift (> 1.5˚ visual angle) was detected at the beginning of each trial, then subjects had to complete a recalibration and revalidation. Events in which velocity was higher than 35°/s and acceleration exceeded 9,500°/s^2^ were recorded as saccades. Microsaccades were detected using the method proposed by Engbert and Kliegl (2003). A microsaccade was defined as saccades with intervals longer than 12 ms and with a velocity above a threshold. The threshold was calculated as a constant λ times the estimated standard deviation of microsaccades velocity distribution within each trial. We chose a value of λ = 6 (Engbert & Kliegl, 2003). Blinks were detected by the eye-tracker as missing pupils during a saccade event. Trials with detected blinks during the presentation of videos (1.1% of all trials) were excluded from the analysis.

Stimuli consisted of 60 videos (about 3-s long) recorded at the University of California Santa Barbara campus across 30 days. The videos were filmed in different settings (classrooms, campus outdoors, student apartments, etc.). In each video, there were between 4–7 people presented. We gave verbal instructions during filming so that multiple people in the video looked simultaneously toward the same person. We manually annotated the start and end times of head and body motions for each gaze-orienting individual by inspecting frame by frame of the videos. The mean start time of heads’ motion was 0.4 s and end time at 1.8 s. The mean start time of bodies’ motion was 0.6 s and end time at 1.7 s (Appendix Fig. 10a–b). To make sure the dynamic gazing behaviors between multiple gaze-orienting people in the same video were mostly synchronized, the standard deviations between the start and end time for heads and bodies in videos with more than one gazing-orienting person were calculated. The mean standard deviations of heads start time (0.05 s) and bodies start times (0.09 s) were both less than 0.1 s. The mean standard deviations of the heads end time and bodies end time were 0.11 s and 0.16 s, respectively (Appendix Fig. 10c–d), indicating a high synchronization of dynamic gaze cueing. In order to ensure the gaze behaviors are natural, we did not give explicit instructions on how to move their heads or/and bodies during filming. All movies had at least one gaze-orienting person turning their heads, and 60% of the movies had at least one gaze-orienting person turning their bodies.

We first extracted individual frames from original videos. Then we used a manual segmentation of individuals’ heads and bodies outlines. To delete target/distractor individuals from the images, we replaced the RGB values of pixels contained by the individual outline in selected frames with the RGB values of those pixels of the immediate background to the individuals. This method allowed us to delete target/distractor individuals from the initial portion of the video frames before the entry of the individuals. Finally, we compiled the processed frames to create videos consisting of (1) one to three individuals orienting their gaze, head, and body towards a point in the scene; (2) the appearance of three/four individuals after 200- or 500-ms delay after completion of the head/body/gaze. In target-present trials (50% of all trials), the target person appeared with two to three distractors. In 50% of target-present trials, the target person appeared at the location where the gaze-orienting individuals looked (valid gaze cue). In the other 50% of the target-present trials, a distractor person instead of a target person appeared at the location where gaze-orienting individuals looked at (invalid gaze cue; Appendix 11a–c). In target-absent trials (50% of all trials), the three or four people that were all distractors appeared (Appendix 11d–f).

**Fig. 1:**
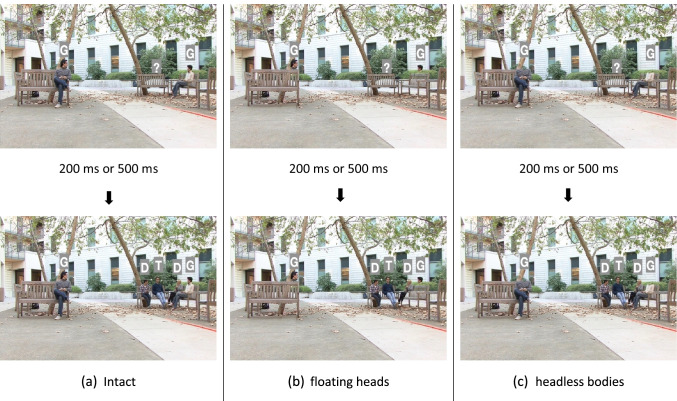
Example frames from videos with valid cues. In the videos, the gaze-orienting individuals (G) look at the target person. After a 200 ms or 500 ms delay, the target person (T) and some other distractor people (D) appeared. (a) The intact condition: Gaze-orienting individuals (G) contain head and bodies; (b) the floating heads condition: gaze-orienting individuals(G) have floating heads without bodies; (c) the headless bodies condition: gaze-orienting individuals (G) have only bodies. All the letter annotations and dashed lines that indicate the gaze directions are just for illustration and were not shown in the actual experiment videos

Figure 1 shows example frames from videos in which the target person was present with valid cues (see Appendix Fig. 11 for more examples from target-present invalid trials and target-absent trials. The reason we chose two or three distractors to appear with the target person was to ensure the task was challenging enough such that the cue validity would affect the behavioral performance. With no distractor, the observer’s attention would simply be attracted to the appearance of the target person in the periphery regardless of the cue validity. Having two or three distractors introduced a reasonable difficulty.

In addition to the intact condition in which gaze orienting individuals’ appeared with heads and bodies, we created videos that selectively deleted gaze orienting individuals’ heads or bodies. The target/distractor individuals appearing after the 200- or 500-ms SOA always contained their entire intact heads and bodies. Thus, there were three experimental conditions: (1) intact videos, (2) floating heads videos (gaze-orienting individuals’ bodies were invisible), and (3) headless bodies videos (gaze-orienting individuals’ heads were invisible). In all videos, we retained the immediate background behind the erased heads or bodies (see Fig. 1).

To verify that the locations in the images of the gaze-orienting people were not predictive of the location of the gazed person, we used a multiple linear regression test using the horizontal positions of the three gaze-orienting people to predict the horizontal location of the gazed person. Results showed the overall regression test was not significant, *F*(3, 42) = 1.12, *R*^2^ = 0.07, *p* = 0.35.

### Behavioral task

**Fig. 2:**
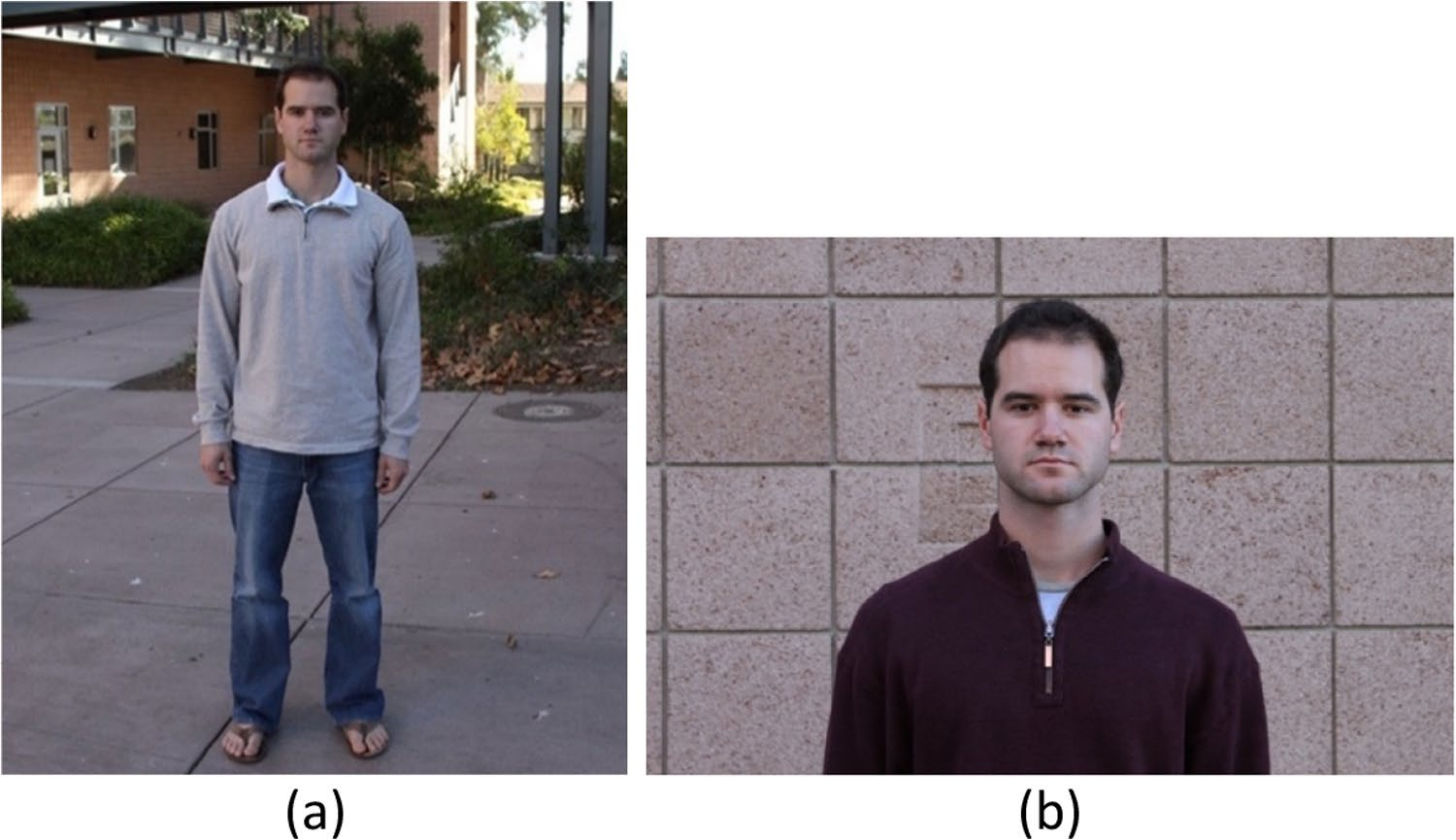
The target person’s photographs. (**a**) Photo of person in standing position, (**b**) Photo with a close-up shot. The target person’s photos were presented on every response screen

Subjects were asked to complete the target detection task while watching videos. Before the practice trials, observers were given unlimited time to familiarize themselves with pictures of the target person in different outfits (see Fig. 2). Then they completed a practice session with 10 videos that were different from the actual experiment videos. Photos of the target person were presented as a reference when they made a response after each video. All participants were able to detect the target above chance after practice.

Participants then completed the main experimental sessions with all three conditions: (1) intact; (2) floating heads; (3) headless bodies. During a session, observers completed all three conditions in random order. Videos were presented in blocks for each condition. Within each condition, 60 different videos were presented randomly. In total, each observer finished 60 trials/condition × 3 conditions/session × 2 sessions = 360 trials.

**Fig. 3:**
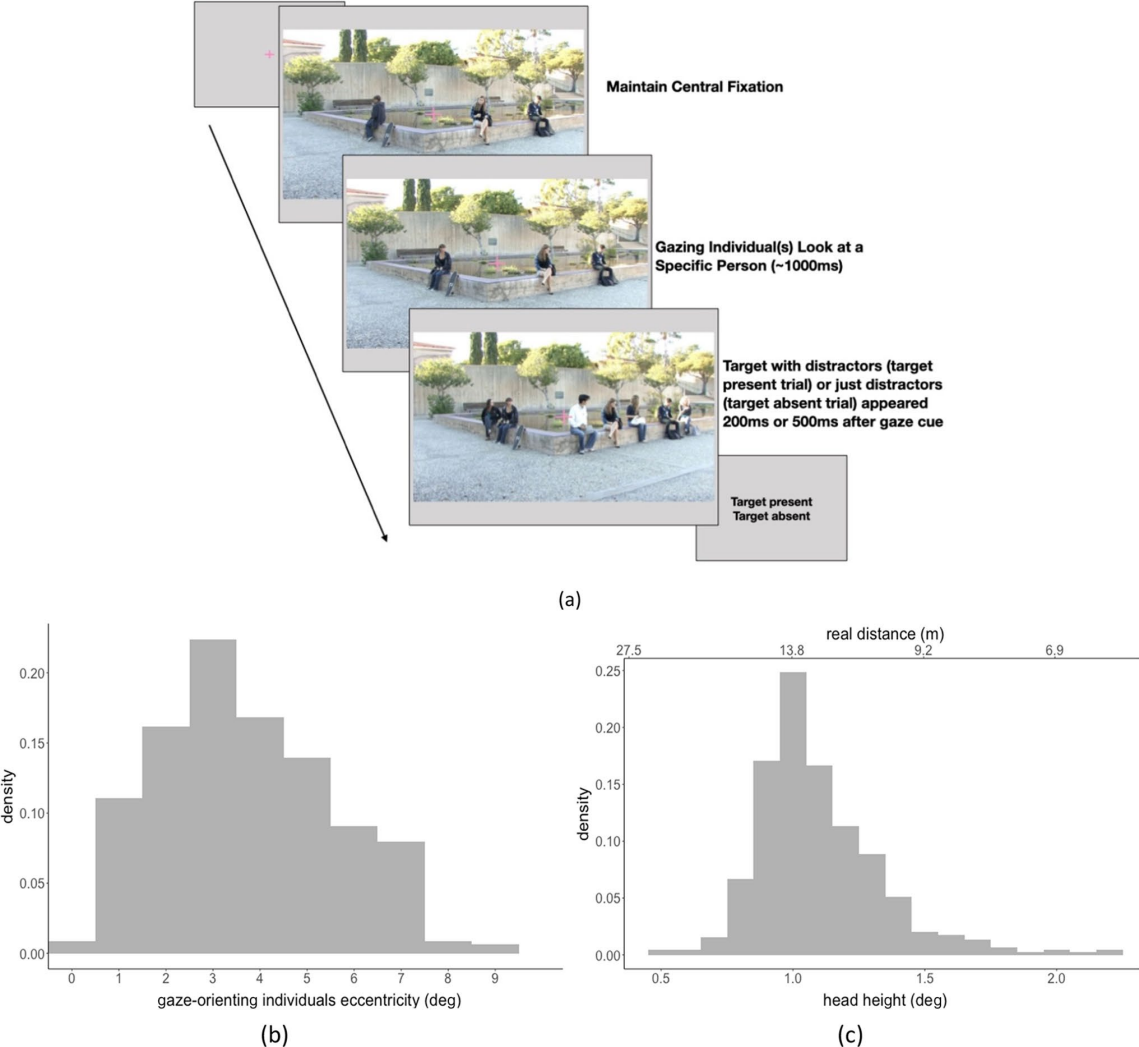
A Timeline for each trial. The participants fixated at the center cross and pressed the space bar to initialize the trial. They maintained fixation at the center cross throughout the video. The trial was aborted if the eye tracker detected a broken fixation (move away from the central fixation by 1.5˚). The video started with the gaze-orienting individuals looking at a common location of a person not visible during that time period. After 200 ms or 500 ms following the end of the gaze-looking behavior, other individuals (target-present trials: target person and distractors; targetabsent trials: only distractors) appeared in the video for 800 ms. Participants indicated whether the target person (50% probability of presence) was present or absent in the video by pressing an upward arrow (present) or a downward arrow (absent). (b) Histogram of retinal eccentricities of the gaze-orienting individuals relative to the central fixation in the movies. (c) Histogram of the vertical size of the gaze-orienting individuals (deg) across all movies. The top-axis is the estimated realworld distances (meters) from the observer that would result in the subtended head vertical angular sizes in the experiment.

Participants first finished a nine-point calibration and validation before experimental sessions started. On each trial, the participants were instructed to fixate a cross at the center of the screen and press the space bar to start the trial. They maintained fixation at the central cross throughout the presentation of the videos. If the infrared video eye tracker detected an eye movement away from the central fixation of more than 1.5˚ visual angle, that trial was aborted. Target and distractors (target-present trials) or only distractors (target-absent trials) were presented for 800 ms, after which a response screen replaced the video. Participants pressed the up arrow or down arrow on the keyboard to indicate if the target person was present or absent (see Fig. 3a). Figure 3b–c show the distribution of retinal eccentricities of all gaze-orienting individuals and the distribution of viewing angles subtended by their heads (vertical size) across all videos. Figure 3c also shows the viewing distances from an observer in the real world that would result in the measured subtended visual angles. We did not control or measure the distances between the camera and the people when we recorded the videos. We estimated the corresponding viewing distance based on typical sizes of people’s heads in the real world, the visual angle that the heads in the images subtended on the observer’s retina, and basic trigonometry. We assumed 0.24 m as the average adult head vertical length (the vertical distance from the bottom of the chin to the top of the head; Lee et al., 2006).

### Data analysis

We first examined the effect of gaze orienting from head and body movement on the subjects’ behavioral performance. We used bootstrap techniques to estimate the statistical significance of variations of hit rate, the difference in sensitivity ∆*d′*, the microsaccades’ amplitude (degrees) toward cue directions, and the proportion of microsaccades toward cue direction.

To apply the bootstrap test, we sampled 30 participants with replacement and sampled all-trial data from the selected 30 subjects (a bootstrap sample) and repeated the process 10,000 times. The distributions of resampled means or mean differences were used to assess statistical significance. All *p* values were corrected using a false discovery rate (FDR) to reduce the probability of making a Type I error.

The procedure was repeated for all three conditions (intact, floating heads, headless bodies), separated by the length of delay of the onset of the target (200 ms vs. 500 ms), and used to evaluate ∆*d′*, microsaccades’ amplitude, and proportion toward cue directions.

## Results

### Quantify gaze information

**Fig. 4:**
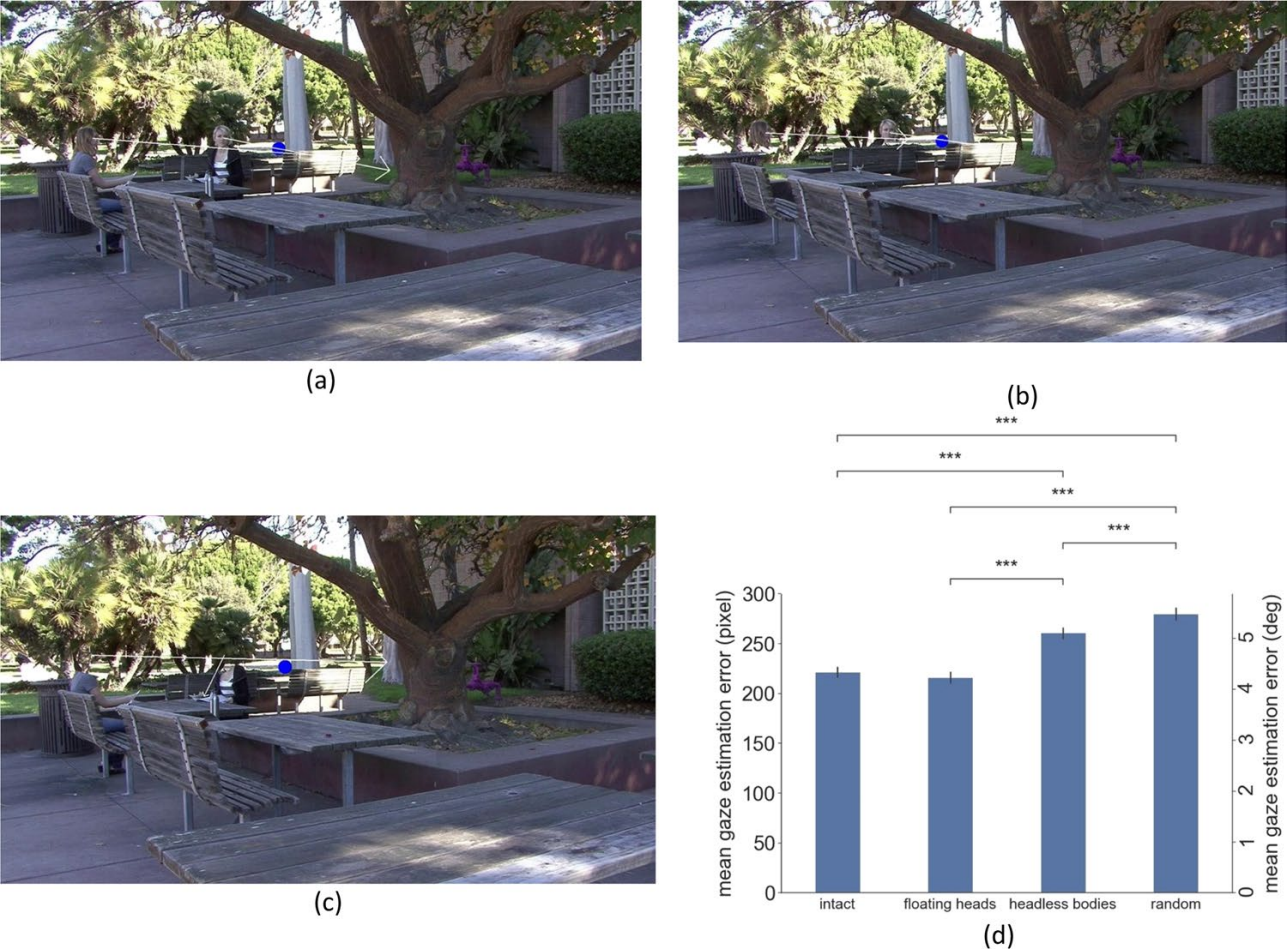
Sample frame we used to calculate the estimation error for the (**a**) intact condition, (**b**) floating heads condition, (**c**) headless bodies condition. The white arrow is the average gaze direction annotated by three research assistants. The blue dot is the ground-truth position of the person they are looking at, who was erased from the video frame. (**d**). mean gaze location estimation error (left: pixel, right: deg) in all conditions and error calculated from random permutations as a control comparison

In order to quantify the gaze information in the intact, floating heads, and headless bodies videos, nine research assistants (three for each condition to avoid memory effects from repeated viewing of a video) manually selected the location of their estimated locus of gaze for all the frames before the target/distractor person appeared in each video. To create a control comparison, we also randomly permuted all the movie frames across trials and calculated the corresponding estimation error. The estimation error was calculated as the Euclidean distance between the ground-truth gazed person’s head location and the mean annotated gazed location. The control condition serves to quantify the estimation expected by chance (Fig. 4).

We found a significantly higher estimation error (diminished gaze information) in the headless bodies condition (mean = 329.9 pixels) compared to both the intact (mean = 152.9 pixels) and the floating heads condition (mean = 137.2 pixels), both *p* < 0.001. No difference between the intact and the floating heads condition was found, *p* = 0.76. Most importantly, the control permuted condition (mean = 241.9 pixels) had a significantly higher estimation error than all conditions, including the headless bodies, all *p* < 0.001. This showed that the headless bodies had lower information about the locus of gaze compared to the other two conditions but still contained some amount of useful gaze information to orient attention.

### Gaze cueing effect on perceptual performance

**Fig. 5:**
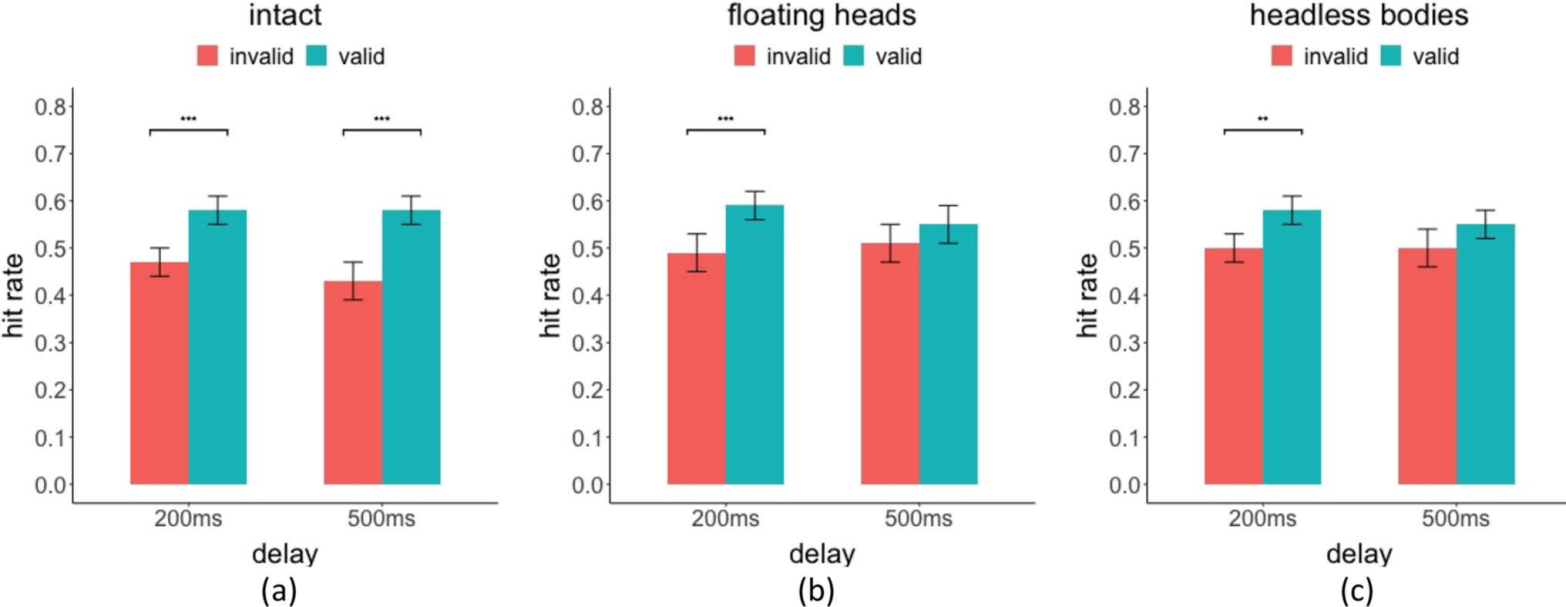
Hit Rate for cue-valid and invalid trials and two SOA delays (200 ms and 500 ms) for (**a**) intact condition, (**b**) floating heads condition, (**c**) and headless bodies condition

We first evaluated the influence of the head/body orienting on the performance of detecting the target person. Table 1 shows the hit rate, false alarm rate, and index of detectability *d′* (Green & Swets, 1989) for each condition and the statistical significance of the cueing effect. Figure 5 shows the hit rates in the three conditions: the intact, the floating heads, and the headless bodies. In the intact condition, valid head/body cues improved accuracy: a significantly higher hit rate (hr) for both the 200 ms SOA (valid hr = 0.58 vs. invalid hr = 0.47, *p* < 0.001, Cohen’s *d* = 0.67, bootstrap for this and all reported tests) and the 500-ms delay (valid hr = 0.58 vs. invalid hr = 0.43, *p* < 0.001, Cohen’s *d* = 0.77). In the floating heads condition, the hit rate was only significantly higher for the valid versus invalid cue trials when the delay was 200 ms (valid hr = 0.59 vs. invalid hr = 0.49, *p* < 0.001, *d* = 0.52), but not with 500-ms delay (valid hr = 0.55 vs. invalid hr = 0.51, *p* = 0.17, *d* = 0.18). Finally, in the headless bodies condition, the cueing effect on hit rate was also found only to be significant in the short delay (hr = 0.58 vs. invalid hr = 0.50, *p* = 0.006, *d* = 0.49), but not in the 500-ms delay (valid hr = 0.55 vs. invalid hr = 0.50, *p* = 0.10, *d* = 0.26). The cueing effect as measured by the hit rate difference (hit rate valid cue − hit rate invalid cue trials) was significantly larger for the intact condition relative to the floating heads and headless bodies for 500 ms (intact vs. floating heads, *p* = 0.007, intact vs. headless bodies, *p* = 0.007; floating heads vs. headless bodies, *p* = 0.60) but not for the 200 ms (intact vs. floating heads, *p* = 0.47, intact vs. headless bodies, *p* = 0.40; floating heads vs. headless bodies, *p* = 0.40). The effect size for the long delay was at least three times larger for the intact condition (*d* = 0.77) relative to the floating heads (*d* = 0.18) and the headless bodies conditions (*d* = 0.26). A similar analysis using *d′* detectability instead of hit rate results in the same findings (Appendix Fig. 12).

**Table 1:** Mean hit rates, *d′*, and false-positive rate, standard errors for each condition in parenthesis, and *p* value from bootstrap resampling tests. The **BOLD** values are significant

	Delay	Cue Validity	Hit Rate	Sensitivity	False Alarm	Cueing Effect
Intact	200	valid	0.58 (0.03)	1.12 (0.11)	0.22 (0.02)	***p* < 0.001**
		invalid	0.47 (0.02)	0.81 (0.11)		
	500	valid	0.58 (0.03)	1.13 (0.16)	0.25 (0.03)	***p* < 0.001**
		invalid	0.43 (0.04)	0.72 (0.17)		
Floating Heads	200	valid	0.59 (0.04)	1.09 (0.14)	0.26 (0.03)	***p* < 0.001**
		invalid	0.49 (0.03)	0.81 (0.14)		
	500	valid	0.55 (0.04)	1.06 (0.17)	0.25 (0.03)	*p* = 0.17
		invalid	0.51 (0.03)	1.01 (0.16)		
Headless Bodies	200	valid	0.58 (0.03)	1.15 (0.11)	0.22 (0.03)	***p* = 0.006**
		invalid	0.50 (0.03)	0.93 (0.12)		
	500	valid	0.55 (0.04)	1.00 (0.16)	0.25 (0.03)	*p* = 0.08
		invalid	0.50 (0.03)	0.93 (0.14)

**Fig. 6:**
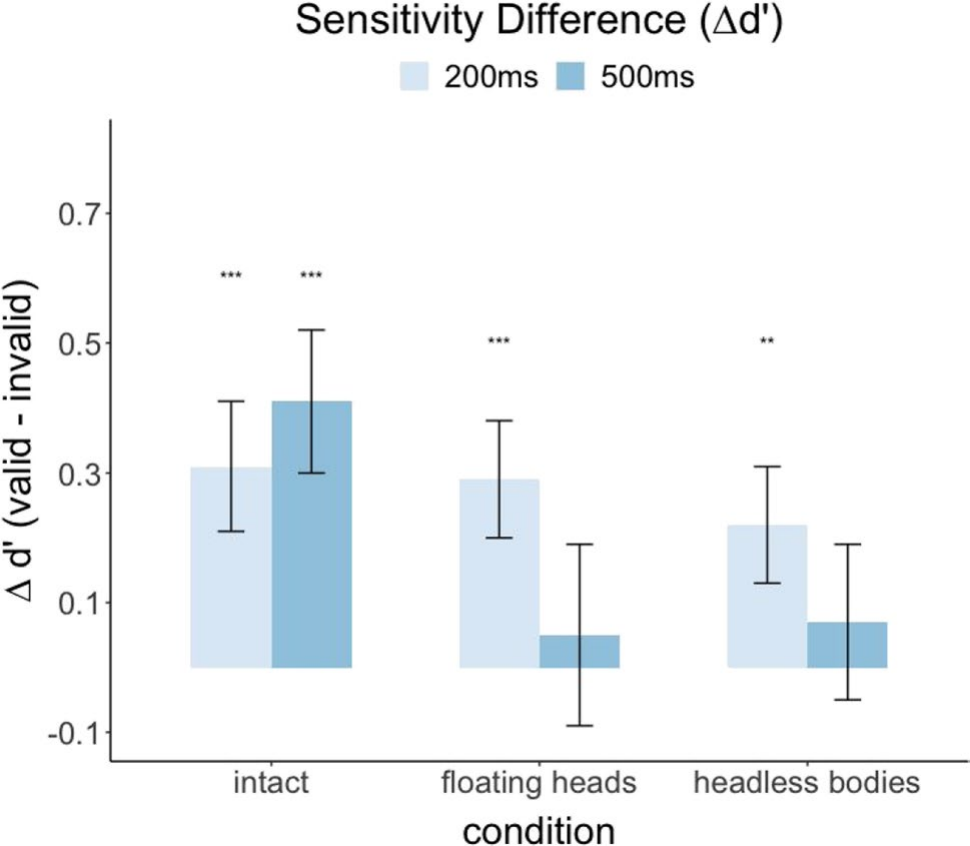
Differences in sensitivity (Δd’) across cue-valid and invalid trials for the three conditions and two SOAs

Figure 6 shows differences in detectability (∆*d′* = valid *d′* − invalid *d′*) across valid and invalid cues for the various conditions. Consistent with hit rate analysis, similar benefits of valid cues were found in the intact condition with both delays (200 ms, *p* < 0.001, ∆*d* = 0.31; 500 ms, *p* < 0.001, ∆*d* = 0.41). For the floating heads and the headless bodies conditions, a significant ∆*d′* was observed with a shorter delay of 200 ms (floating heads 200 ms, *p* < 0.001, ∆*d* = 0.29; headless bodies 200 ms, *p* = 0.006, ∆*d* = 0.22, respectively) but not the longer delay (floating heads 500 ms, *p* = 0.36, ∆*d* = 0.05; headless bodies 500 ms, *p* = 0.33, ∆*d* = 0.07). The results suggest that the cueing effects elicited by floating heads or headless bodies develop quickly but do not sustain and diminish for the longer SOA delay (500 ms). In addition, the ∆*d′* across valid and invalid cues for the 500 ms was significantly higher for the intact relative to the floating heads, *p* = 0.007, and headless bodies conditions, *p* = 0.006. The ∆*d′*s were not significantly different for the 200 ms between the conditions (intact vs. floating heads, *p* = 0.43, intact vs. headless bodies, *p* = 0.40; floating heads vs. headless bodies, *p* = 0.40). We also tested whether the number of gaze-orienting people (number of cues) influenced the cueing effect (∆*d′*) and did not find a significant effect, all *p*s > 0.05.

The response times in our study were not as informative as the behavioral performance because we gave unlimited time to participants to make a response after each video. A within-subject three-way ANOVA (condition × cue validity × SOA) on response time resulted in no significant main effect of condition, *F*(2, 58) = 0.21, *p* = 0.82, cue validity, *F*(2, 58) = 2.57, *p* = 0.08, or SOA, *F*(1, 29) = 2.11, *p* = 0.16.

Cueing effects on microsaccade direction.

**Fig. 7:**
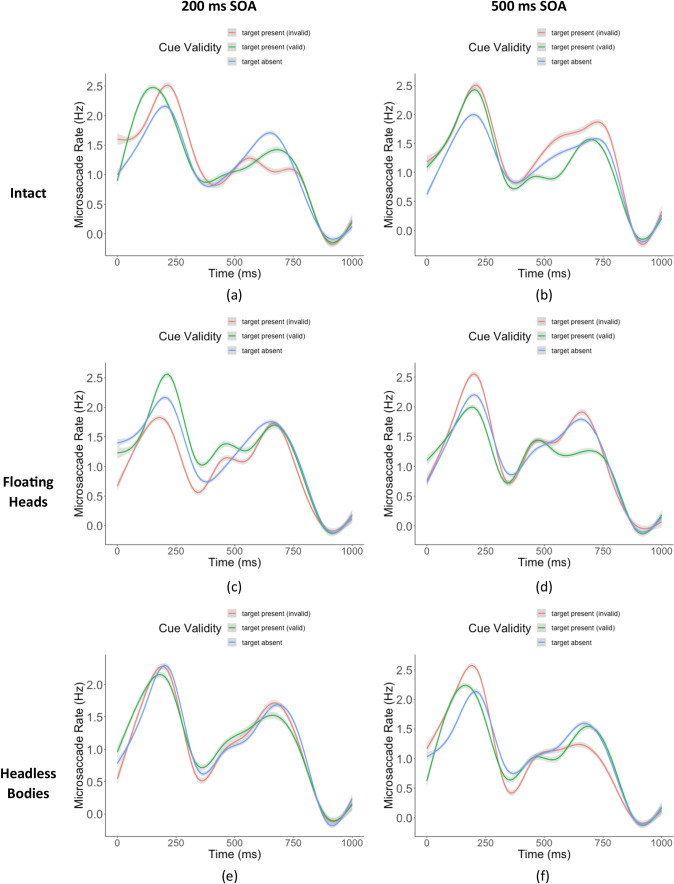
Microsaccade rate starting from video in three conditions with different SOAs. Intact (**a**)-(**b**) floating heads (**c**)-(**d**), headless bodies (**e**)-(**f**), the left column is 200 ms SOA, the right column is 500 ms SOA, the shaded area is 95% confidence interval across subjects. Time t = 0 represents the video onset

Figure 7 shows microsaccade rates (Rolfs et al., 2008) starting with the video onset. Because the timing of cueing dynamics was different across trials, we computed the microsaccade rates for a period of 1,000 ms aligned with each video’s onset. The pattern of microsaccades was similar across three conditions, with a peak around 200 ms from video onset and a decrease later around 300 ms. Aligning eye movement data relative to the onset of the head motion of each video resulted in slightly noisier patterns in different conditions with a peak around 400–500 ms (Appendix Fig. 13).

We analyzed the amplitude and direction of microsaccades of the time interval before and during the target presentation. Overall, the distribution of microsaccade amplitudes was consistent with previous results (Laubrock et al., 2010; Martinez-Conde et al., 2009). Across the entire timecourse, 79.6% of the microsaccade amplitudes were less than 0.5˚ visual angle (histogram in Appendix Fig. 14). The median microsaccade amplitude was significantly higher when both head and body were presented (intact median = 15.6’) than either only heads (floating heads median = 14.9’, ${\chi }^{2}$= 6.06, *p* = 0.02, effect size ϕ = 0.02) (Kim, 2017), or only bodies were present (headless bodies median = 14.2’, ${\chi }^{2}$= 24.29, *p* < 0.001, ϕ = 0.04). The median microsaccades amplitude for the floating heads condition was the same as the headless bodies condition (${\chi }^{2}$= 3.31, *p* = 0.06, ϕ = 0.01).

**Fig. 8:**
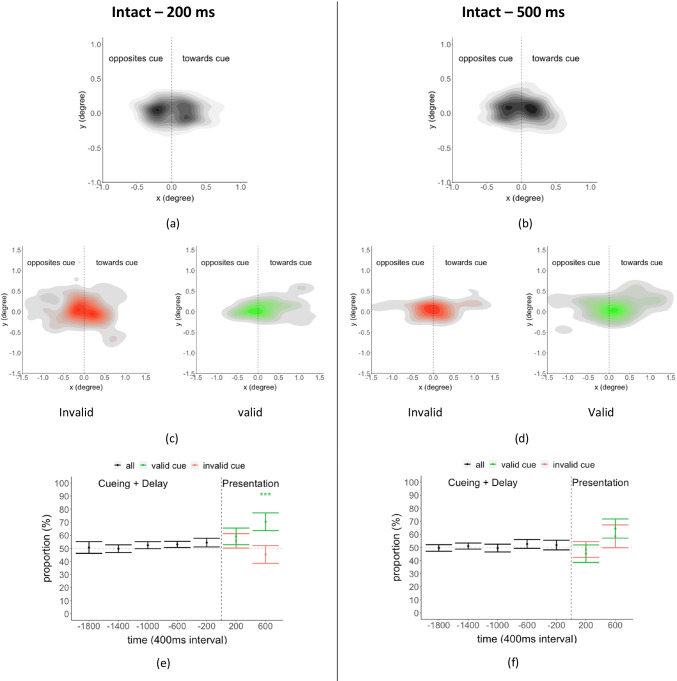
Microsaccades for the intact condition. (**a**)-(**b**) Heatmaps of microsaccades for the period before the target/distractors presentation with black and white colors (a darker color corresponds to higher density). (**c**)-(**d**) Heatmaps of microsaccades during 400–800 ms after target/distractor presentation (red color for invalid trials, green color for valid trials). (**e**)-(**f**) Proportion of microsaccades that moved toward the cued direction as a function of time for intact condition 200 ms and 500 ms delay over time. The x-axis indicates the temporal midpoint of the time window (200 represents the time window 0-400 ms, etc.). Time t = 0 is lined up with respect to target and distractor presentation. The y-axis is the mean proportion of microsaccades that moved toward the cue direction

We plotted heatmaps to show the density of microsaccade directions and amplitudes (Fig. 8a–d) aligned to the gaze-cue direction. Figure 8a–b shows the microsaccade density before the presentation of the target/distractors for the 200 ms and 500 ms SOAs. Figures 8c–d show the density plots 400 ms to 800 ms after the presentation onset of the target/distractor separately for valid and invalid cue trials (for 200 ms and 500 ms SOA).

To quantify the effect of gaze cueing on microsaccade direction, we calculated the average proportion of microsaccades per trial moving toward the cued direction (left/right of the central fixation) using a 400-ms window. Figure 8e–f shows the proportion of microsaccades toward the cued direction for the intact condition for the 200-ms and 500-ms conditions (see Appendix Figs. 15-16 for the floating heads and headless bodies conditions). We separately computed the measure for the time period before and during the presentation of targets/distractors. We found a significant microsaccade direction bias in the valid trials from the intact and the floating heads condition (SOA 200 ms) during the period 400 ms to 800 ms after the target/distractors onset. This indicates that the presence of heads in dynamic gaze cueing is necessary to trigger microsaccades toward the cued direction with a short SOA delay. Furthermore, a 200 ms SOA indicated that the effect of microsaccade direction bias peaked around 600–1,000 ms (400 ms to 800 ms + 200 ms) after the dynamic gaze cue completion.

**Fig. 9:**
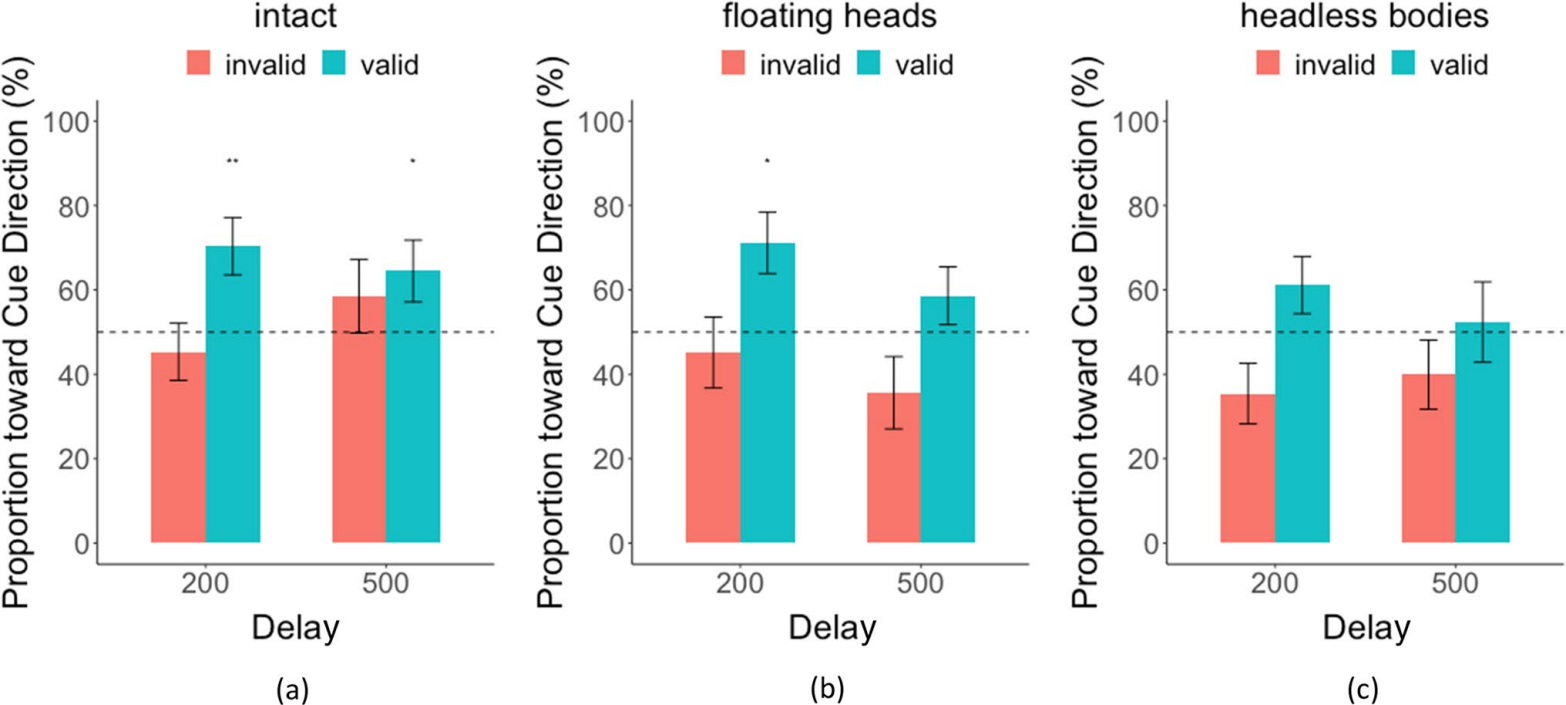
The proportion of microsaccades that moved toward the cue direction within 400 ms-800 ms after target/distractors presentation onset for (**a**) intact condition, (**b**) floating heads condition, and (**c**) headless bodies condition. Results are shown for the two SOAs

Figure 9 summarizes the proportion of microsaccades toward the cued direction just during the period of 400 ms to 800 ms after targets/distractors onset for the three conditions: the intact, the floating heads, and the headless bodies. Table 2 summarizes the proportion of microsaccades and degrees toward the cued direction for the period of 400 ms to 800 ms after targets/distractors onset: valid vs. invalid trials for the two SOAs and three conditions (intact, floating heads, headless bodies). Consistent with the behavioral performance (Fig. 5), we found a significant bias towards the cue direction for the valid trials in the intact head/body condition for both 200 ms and 500 ms (200 ms, *p* = 0.005, *d* = 0.75; 500 ms, *p* = 0.047, *d* = 0.52). We also found a significant bias towards the cue direction for the valid trials for floating heads condition only with an SOA of 200 ms (*p* = 0.03, *d* = 0.58; see Table 2 & Fig. 9) but not with an SOA of 500 ms (*p* = 0.19, *d* = 0.24). There were no significant effects on microsaccades towards the valid cue direction for the headless bodies condition for either SOAs (200 ms, *p* = 0.10, *d* = 0.40; 500 ms, *p* = 0.46, *d* = 0.05). We also quantified the average microsaccades’ degrees toward the cued direction for valid and invalid trials over time and summarized the result in Table 2 (See also Appendix Figs. 17-18).

**Table 2:** The average microsaccades visual angle degrees toward the cue direction. The **BOLD** values are significant

Condition	SOA	Cue Validity	Proportion toward Cue direction	Degree toward Cue direction
Intact	200	valid	**70.34% (*p* = 0.005)**	**0.23˚ (*p* = 0.001)**
		invalid	45.33% (*p* = 0.30)	− 0.13˚(*p* = 0.37)
	500	valid	**64.46% (*p* = 0.047)**	0.05˚ (*p* = 0.27)
		invalid	58.51% (*p* = 0.21)	0.02˚ (*p* = 0.37)
Floating Heads	200	valid	**71.12% (*p* = 0.03)**	**0.20˚ (*p* = 0.002)**
		invalid	45.16% (*p* = 0.49)	− 0.09˚ (*p* = 0.80)
	500	valid	58.61% (*p* = 0.19)	0.10˚ (*p* = 0.06)
		invalid	35.61% (*p* = 0.13)	− 0.12˚ (*p* = 0.80)
Headless Bodies	200	valid	61.14% (*p* = 0.10)	0.03˚ (*p* = 0.31)
		invalid	35.42% (*p* = 0.13)	− 0.06˚ (*p* = 0.99)
	500	valid	52.38% (*p* = 0.46)	0.08˚ (*p* = 0.31)
		invalid	39.92% (*p* = 0.16)	− 0.06˚ (*p* = 0.99)

The sustained influence of the joint presence of head and body in orienting attention is also shown for the 500-ms SOA by the trend of a microsaccade bias even for invalid cue trials (Fig. 9a). This trend is not present for the 500-ms SOA invalid cue trials of the floating heads or headless bodies conditions (although bootstrap tests with FDR correction did not reach statistical significance when comparing intact vs. the average of the two other conditions, *p* = 0.057, *d* = 0.40).

## Discussion

Our goal was to measure how gaze, heads, and bodies during natural behaviors contribute to orienting attention in an ecological search task with dynamic scenes. The majority of previous studies with simpler drawings, point-light stimuli (Shi et al., 2010), and simple videos of heads suggest that gaze, head, and body direction result in cueing effects that persist up to 600 ms or beyond. However, in all of these studies, the gaze/posture cue is presented foveally and subtends a large viewing angle. Our study found a different result: heads and bodies oriented attention in a sustained manner until 500 ms only when presented jointly. The separate presentations of the head or body led to more transient cueing effects, which diminished with the 500-ms delay. What factors might explain the discrepancy from previous results? We aimed to simulate a broader range of scenarios in the real world where the observer views a scene from medium to large distances and gazing individuals appear at various retinal eccentricities. Previous studies have shown that at distances beyond 4–5 degrees, inferences about gaze direction from eye orientation are highly degraded (Loomis et al., 2008), and cueing gaze effects are reduced (Yokoyama & Takeda, 2019). As retinal eccentricity increases, head orientation plays a larger role as information about eye orientation becomes less accessible due to crowding in the visual periphery (Florey et al., 2015). The more difficult access to eye orientation might explain the difference between the current and previous results. In the present study, we could not isolate the specific relationship between the eccentricity of gaze-orienting people and observer performance because each video presented multiple individuals at different eccentricities.

We integrate our findings into the current literature as follows. When the eyes are presented foveally and clearly visible, gaze direction generates a sustained orienting of attention. In situations in which the individuals are more distant and away from the point of fixation, sustained attention requires the presence of the whole head and body of the gazing individuals. Our results also suggest that the contributions to the orienting of covert attention of the head, body, and their joint presence might be related to the inherent information of the cues about the locus of gaze. Cues that provide higher information about the location of a target might have a larger influence on orienting covert attention. Whole-intact bodies oriented covert attention the most and also contained the most information about the location of gaze as assessed by explicit human location judgments. The headless bodies oriented covert attention the least and also contained the least inherent information about the locus of the gaze. This relationship between cues and the orienting of attention is also present in search of scenes. Objects co-occurring with a target orient overt attention more than scene backgrounds and also provide more precise information about likely target locations (Koehler & Eckstein, 2017a, b).

Where might the integration of the various gaze, head, and body parts occur? Neuropsychological and neuroimaging studies point to the superior temporal sulcus (STS) playing an important role in integrating multiple cues to develop social perception. STS cells are sensitive to images of the face, gaze direction, mouth movement, head orientation (Perrett et al., 1985, 1992), biological motion such as eyes, mouth, and body movements (Bonda et al., 1996; Grossman et al., 2000), and goal-oriented hand and body movements that help infer another person’s attention (Bonda et al., 1996; Jellema et al., 2000; Perrett et al., 1989). Humans and monkeys with damaged STS regions show difficulty identifying others’ faces, gaze direction, and intention (Campbell et al., 1990; Heywood & Cowey, 1992).

Our study also investigated the effect of the direction of the gazing individuals on microsaccades. Previous studies found microsaccades direction biases toward endogenous cue (e.g., central cue) direction around 200 ms to 400 ms after cue onset during a saccade rate rebound period (Engbert & Kliegl, 2003). However, with exogenous cues (peripheral flash), microsaccade directions were found to be biased toward cue direction around 20 ms to 200 ms after cue onset, then shift back to the opposite direction around 600 ms to 800 ms (Laubrock et al., 2005; Rolfs et al., 2004). We found an amplitude of the microsaccade bias toward cue direction (~ 0.2˚) that is higher than previous studies (less than 0.05˚) (Meyberg, Sinn, et al., 2017; Meyberg, Sommer, et al., 2017). Yet, our current results differ from previous studies in important ways. The dynamic cueing by the combination of heads and bodies resulted in a microsaccade direction bias toward the cue direction at a much later time period, around 800–1,000 ms after the completion of the movement of the gazing individuals. The longer delays might be related to (1) the cues in our study were not static but dynamic social cues which developed throughout the video; (2) the dynamic cues were not at the center but in the periphery; (3) we presented multiple gaze-cueing individuals. Thus, it might take a longer time to integrate peripheral information across the various gazing individuals. The microsaccades also showed more frequent movements towards the cue direction in valid vs. invalid trials for the intact condition but not for the headless bodies condition.

In addition, we did not find the typical microsaccade inhibition, which happened around 100 ms immediately after synthetic cue onsets (Engbert & Kliegl, 2003; Rolfs et al., 2008). The dynamic nature of the gaze cues, instead of the static cue in other studies, is most likely the reason for the nontypical microsaccade rate pattern.

Our study found consistency between the orienting of covert attention as measured by the influence of the cue on perceptual performance and by the microsaccade shifts. Microsaccade direction biases and cue validity benefits on behavioral performance were present for an SOA of 200 ms after cue presentation for the intact and floating heads conditions. Only the intact condition showed sustained effect with an SOA of 500 ms for both behavioral performance and microsaccade direction bias. The headless bodies showed a higher behavioral performance but did not show a significant microsaccade direction bias with an SOA of 200 ms. Together, the results are consistent with previous evidence and interpretations suggesting that microsaccades precede or reflect shifts of covert attention (Engbert & Kliegl, 2003; Hafed & Clark, 2002; Laubrock et al., 2010; Yuval-Greenberg et al., 2014).

One limitation in our interpretation is that we have discussed the performance differences across valid/invalid trials solely in terms of a benefit for valid gaze trials. For synthetic cues, a comparison of the invalid cue condition relative to a neutral cue condition suggests a cost for invalid cue trials (Gawryszewski et al., 1987; Posner, 1980; Thiel et al., 2004; Wright et al., 1995). Thus, it might be the case that there is also a cost for gaze invalid trials. Testing such a hypothesis would require implementing a neutral gaze condition for these real-world videos. One possibility is to film scenes with the absence of the gazers and compare target detection relative to the same scenes with invalid gaze trials.

Finally, our study provides an extension of previous studies to more ecologically valid tasks. A majority of studies measure facilitation in the gaze direction for the detection of very simple stimuli. Our study demonstrates the role of gaze, head, and body movements in orienting attention and its impact on decision accuracy for a complex and real-world task such as a person search in cluttered scenes. The experimental framework and publicly available videos can be potentially used to study individuals with deficits in social attention (Chawarska & Shic, 2009; Freeth et al., 2010; Klin et al., 2002; Pierno et al., 2006; Ristic et al., 2005) using a paradigm that better reflects real-world scenes and tasks.

## Figures and Tables

**Fig. 10:**
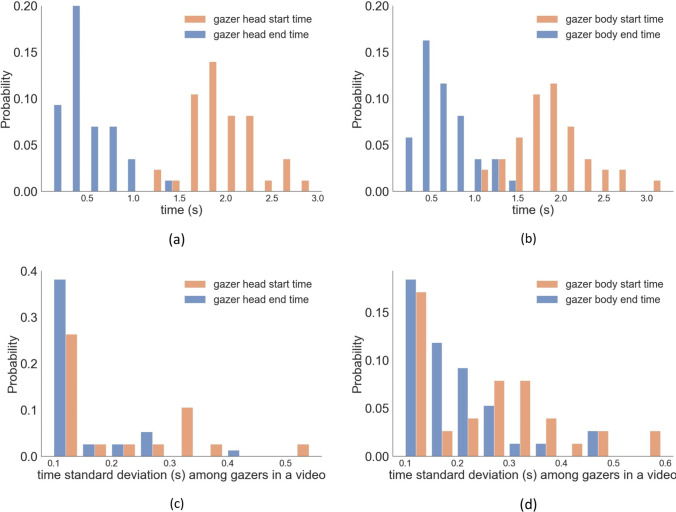
**a–b** Histogram of the starting/ending time of head and body motion across all movies. **c–d** Histogram of standard deviations of starting/ending time among head/body motion across multiple gazers within each video

**Fig. 11:**
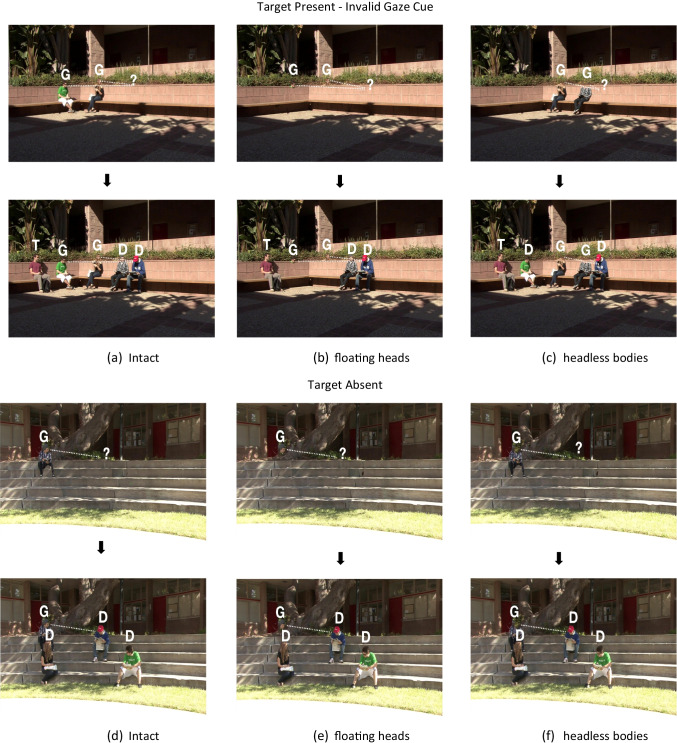
Example frames from three conditions. Gaze-orienting people (G), Target (T), Distractor (D). **a–c** Target present with an invalid cue: Gaze-orienting people (G) gaze at the location with the target person (T), and two distractors (D) appeared after a 300-ms or 500-ms delay. In this case, a distractor (D) instead of the target (T) appeared at the gazed-at location, so the gaze cue was invalid. **d–f** Target absent: Gaze-orienting people (G) looked at the location with three distractors (D) which were presented after a 300-ms or 500-ms delay. All the letter annotations and dashed lines that indicate the gaze directions were not included in the actual experiment videos

**Fig. 12:**
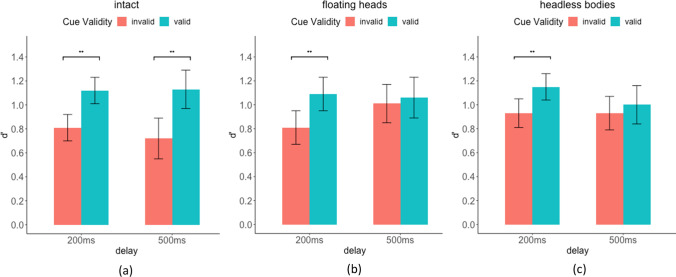
d′ (sensitivity) in all three conditions and two delays

**Fig. 13:**
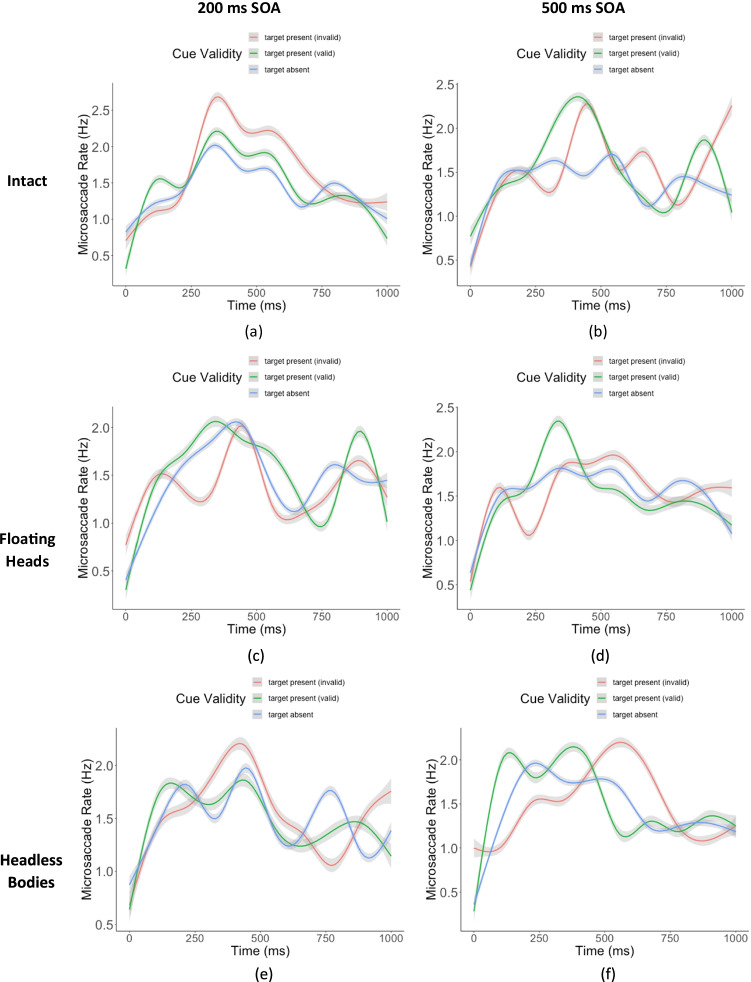
Microsaccade rate starting from the head cue movement in three conditions with different SOAs. **a–b** Intact, **c–d** floating heads, **e–f** headless bodies, the left column is 200-ms SOA, the right column is 500-ms SOA, the shaded area is 95% confidence interval across subjects. Time 0 represents the starting of the head motion

**Fig. 14:**
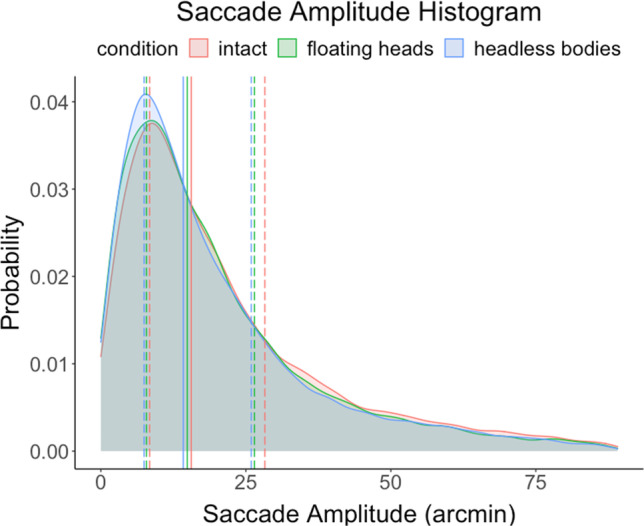
Microsaccade amplitude histogram in all three conditions. 79.64% of all microsaccades were less than 30 arcmins (0.5˚). Vertical lines are medians, and dashed lines are 25%-75% interquartile for each condition

**Fig. 15:**
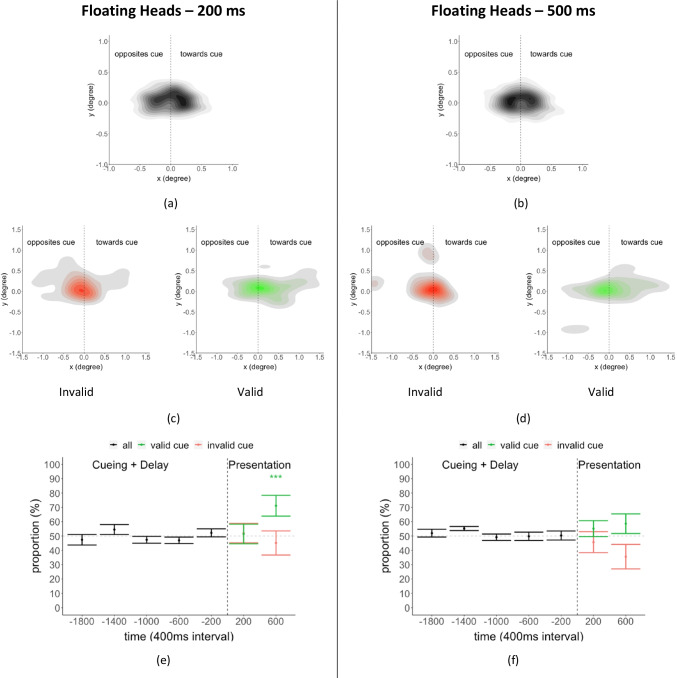
Condition floating heads with 200-ms and 500-ms SOA. **a–b** Heatmaps of microsaccades for the period before the target/distractors presentation with black and white colors (a darker color corresponds to higher density). **c–d** Heatmaps of microsaccades during 400–800 ms after target/distractor presentation (red color for invalid trials, green color for valid trials). **e–f** Proportion of microsaccades that moved toward the cued direction as a function of time for intact condition 200-ms and 500-ms delay over time. The *x*-axis indicates the temporal midpoint of the time window (200 represents the time window 0–400 ms, etc.). Time t = 0 is lined up with respect to target and distractor presentation. The *y*-axis is the mean proportion of microsaccades that moved toward the cue direction

**Fig. 16:**
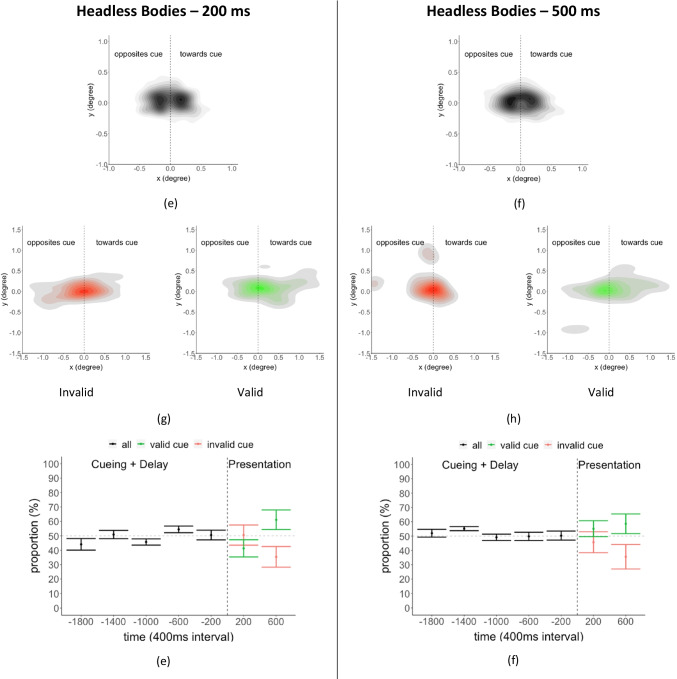
Condition headless bodies with 200-ms and 500-ms SOA. **a–b** Heatmaps of microsaccades for the period before the target/distractors presentation with black and white colors (a darker color corresponds to higher density). **c–d** Heatmaps of microsaccades during 400–800 ms after target/distractor presentation (red color for invalid trials, green color for valid trials). **e–f** Proportion of microsaccades that moved toward the cued direction as a function of time for intact condition 200-ms and 500-ms delay over time. The *x*-axis indicates the temporal midpoint of the time window (200 represents the time window 0–400 ms, etc.). Time t = 0 is lined up with respect to target and distractor presentation. The *y*-axis is the mean proportion of microsaccades that moved toward the cue direction

**Fig. 17:**
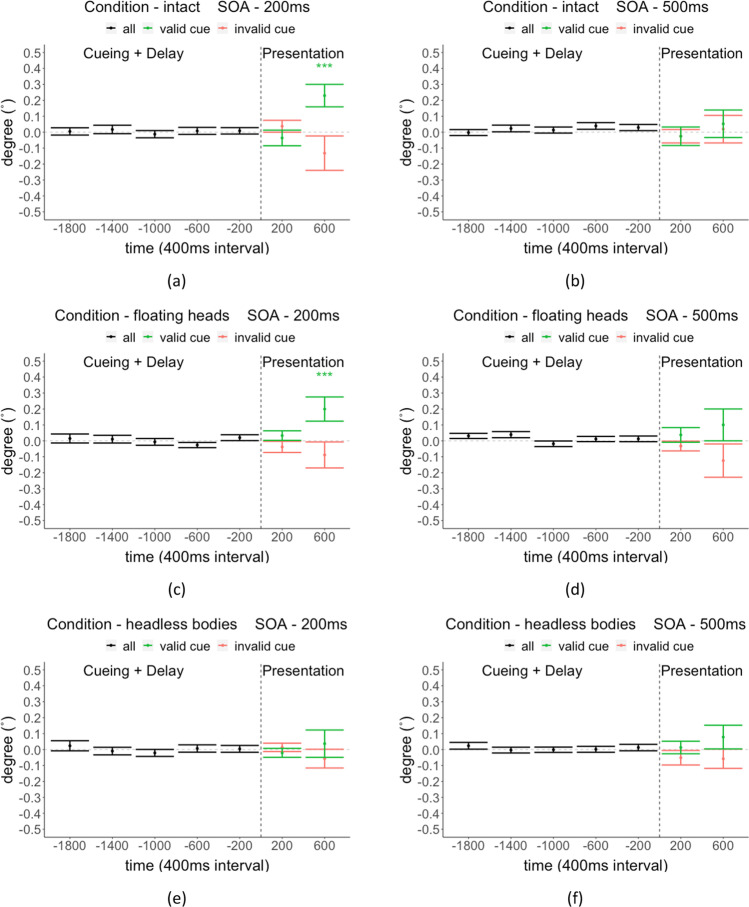
Microsaccades degrees toward the cued direction (left/right side of forced fixation) as a function of time for the **a–b** intact condition at 200 ms and 500 ms, **c–d** the floating heads condition, and **e–f** the headless bodies condition. The *x*-axis indicates the temporal midpoint of the time window (200 represents the time window 0–400 ms, etc.). The *y*-axis is the mean deflection in degrees in visual angle toward the cue direction (positive values represent that the microsaccades are biased toward the cue direction). Black bars represent microsaccades before target/distractors presentation averaged across both valid and invalid trials. Green and red bars represent microsaccades after target/distractors presentation for valid and invalid trials, respectively

**Fig. 18:**
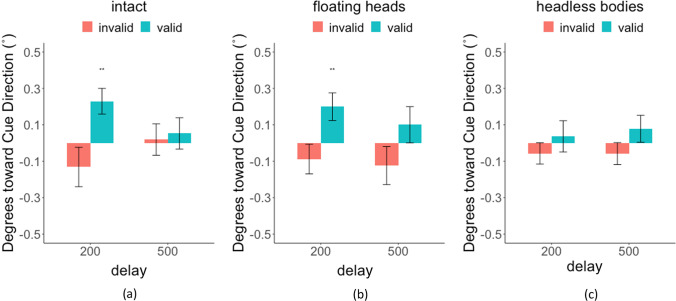
Microsaccades degrees toward the cued direction summarized within 400-ms to 800-ms after target/distractors presentation onset for (**a**) intact condition, (**b**) floating heads condition, (**c**) and headless bodies condition

## Data Availability

The data, video stimuli, and scripts are available to the public (https://osf.io/g7rq6/).

## Appendix

Please see Figs. 10, 11, 12, 13, 14, 15, 16, 17 and 18

## Declarations

### Open practice statement

The experiment was not preregistered. The data are available to the public.
